# High-affinity ELR+ CXC chemokines display balanced G protein-, GRK- and β-arrestin-associated transducer engagement at the viral G protein-coupled receptor ORF74

**DOI:** 10.3389/fphar.2026.1953308

**Published:** 2026-09-25

**Authors:** Qianqian Kong, Brent Van Bosstraeten, Steven De Jonghe, Dominique Schols, Tom Van Loy

**Affiliations:** Department of Microbiology, Immunology and Transplantation, Rega Institute for Medical Research, Molecular, Structural and Translational Virology Research Group, KU Leuven, Leuven, Belgium

**Keywords:** ELR+ CXC chemokines, G protein, GRK, ligand bias, NanoBiT, ORF74, transducer engagement, β-arrestin

## Abstract

Open reading frame 74 (ORF74) is the single G protein-coupled receptor (GPCR) encoded in the genome of Kaposi’s sarcoma-associated herpesvirus (KSHV). Due to its high constitutive activity and broad chemokine binding profile, ORF74 contributes to KSHV-associated diseases through autocrine and paracrine mechanisms that promote cell proliferation, inflammation and angiogenesis. Previous studies showed that ELR+ CXC chemokines (i.e., CXCL1–3 and CXCL5–8), which contain a conserved Glu-Leu-Arg motif, bind ORF74 with high affinity. Among these chemokines, CXCL1–3 and CXCL6–8 act as full or partial agonists in ORF74-mediated G protein signaling and β-arrestin recruitment, whereas CXCL5 behaves as a neutral ligand. However, whether these chemokines preferentially engage specific intracellular transducers remains unclear. We therefore applied a unified NanoLuc Binary Technology (NanoBiT) platform in HEK293A cells to systematically compare how ELR+ CXC chemokines modulate ORF74–transducer proximity across the G protein–GRK–β-arrestin axis. CXCL1–3 and CXCL6–8, but not CXCL5, induced concentration-dependent ORF74-specific responses involving miniG_i_ recruitment, Gβ1 proximity, GRK2/3/5/6 proximity and β-arrestin1/2 recruitment. Using CXCL1 as the reference ligand, bias analysis showed that the active ELR+ CXC chemokines engage ORF74 in a largely balanced manner across these proximal transducer readouts.

## Introduction

G protein-coupled receptors (GPCRs) are membrane proteins that mediate cellular responses to diverse extracellular stimuli, including hormones, neurotransmitters, peptides, lipids and sensory cues. Upon activation, GPCRs engage intracellular signaling machinery, most notably heterotrimeric G proteins (Gαβγ), G protein-coupled receptor kinases (GRKs) and β-arrestins, thereby initiating complex signaling networks that regulate physiological processes (Hilger et al., 2018). Accumulating evidence demonstrates that GPCRs can adopt multiple active conformations, each enabling distinct engagement with signal transducers and resulting in different signaling outcomes. This dynamic nature of GPCR conformations supports the concept of ligand bias, also referred to as biased agonism, since different ligands may stabilize distinct conformations of the same receptor and thus promote preferential coupling to particular G proteins, GRKs or β-arrestins (Urban et al., 2007).

Ligand bias is particularly relevant for promiscuous signaling systems. Human chemokines are small proteins that, through interaction with their cognate GPCRs, regulate immune cell migration and activation. Deregulation of the chemokine–chemokine receptor signaling axis is associated with many inflammatory diseases and cancer (Nagarsheth et al., 2017; Hughes and Nibbs, 2018). Human chemokine receptors frequently interact with multiple chemokines, which may elicit ligand-specific signaling outputs (Eiger et al., 2021). For several receptors, such as CXCR1 (Boon et al., 2025; Jassal et al., 2026), CXCR2 (Jassal et al., 2026; Boon et al., 2024; Saha et al., 2025), CXCR3 (Zheng et al., 2022; Gardner et al., 2024), and CCR7 (Jørgensen et al., 2018; Vanalken et al., 2022), ligand-dependent differences in G protein activation, GRK and/or β-arrestin recruitment, and receptor trafficking have been reported. These findings highlight the prevalence of biased signaling within the chemokine receptor family and suggest that it may serve as an additional layer of chemokine receptor regulation.

Several human herpesviruses have acquired chemokine receptor homologues in their genomes during co-evolution with their hosts. Expression and activity of these receptors support viral persistence and dissemination and contribute to infection-related pathogenesis (De Groof et al., 2021; Rosenkilde et al., 2022; Tsutsumi et al., 2025). Open reading frame 74 (ORF74) is the single GPCR encoded by Kaposi’s sarcoma-associated herpesvirus (KSHV), also known as human herpesvirus 8 (HHV8) (Cesarman et al., 1996). ORF74 is most closely related to the human chemokine receptor CXCR2, displays high constitutive activity and interacts with a broad ligand repertoire, comprising at least fourteen chemokines from the CXC and CC subfamilies that modulate receptor activity as agonists, inverse agonists or antagonists (Rosenkilde et al., 1999; Kong et al., 2025; 2026). In particular, ELR+ CXC chemokines (i.e., CXCL1–3 and CXCL5–8), which contain a conserved Glu-Leu-Arg motif, bind ORF74 with high affinity and, with the exception of CXCL5, act as ORF74 agonists (Kong et al., 2025). Among them, CXCL1 displays the highest apparent affinity for ORF74, and recent cryo-EM structures of CXCL1-bound ORF74–Gi complexes have revealed its ligand-binding mode and Gi-coupled active conformation (Liu et al., 2024; Park et al., 2025).

This broad and functionally diverse ORF74 ligand repertoire raises the possibility that individual chemokines may stabilize distinct receptor conformations, differentially regulate downstream signaling pathways and fine-tune ORF74-mediated signaling (Tsutsumi et al., 2025). However, it remains unclear whether individual ligands exhibit genuine signaling bias through preferential engagement of specific intracellular transducers, as observed for human chemokine receptors (Eiger et al., 2021). To address this question, we employed a unified NanoLuc Binary Technology (NanoBiT) platform in a common cellular background to investigate ORF74-associated transducer responses induced by ELR+ CXC chemokines. This analysis confirmed that CXCL1–3 and CXCL6–8 activate ORF74 across miniG_i_, Gβ1, GRK2/3/5/6 and β-arrestin1/2 readouts, but revealed no significant ligand bias relative to CXCL1, indicating largely balanced ORF74 engagement across the G protein–GRK–β-arrestin transducer axis.

## Materials and methods

### Chemokines, reagents and plasmids

Recombinant human CXCL1 (#300-11), CXCL2 (#300-39), CXCL3 (#300-40), CXCL5 (#300-22B), CXCL6 (#300-41), CXCL7 (#300-14), and CXCL8 (#200-08M) were purchased from PeproTech. The pcDNA3.1(+)-ORF74 plasmid was generated in-house as described previously (Kong et al., 2025). ORF74-LgBiT and ORF74-SmBiT were constructed from pcDNA3.1(+)-ORF74 by fusing LgBiT or SmBiT, respectively, to the C terminus of ORF74 through a linker (GNSSSGGGGSGGGGSSG). SmBiT-Gβ1, SmBiT-β-arrestin1, and SmBiT-β-arrestin2 were constructed in the pEF1α-IRES vector by fusing SmBiT to the N terminus of Gβ1, β-arrestin1, or β-arrestin2, respectively, through a linker (GSSGGGGSGGGG). miniG_i_ is an engineered GTPase domain of the Gα_i_ subunit (Wan et al., 2018). LgBiT-miniG_i_ and LgBiT-GRK2/3/5/6, which carry an N-terminal LgBiT tag, were kindly provided by Dr. Andy Chevigné (Luxembourg Institute of Health) (D’Uonnolo et al., 2022; Palmer et al., 2022).

### Cell culture

HEK293A cells were kindly provided by Prof. A. Inoue (Tohoku University, Sendai, Japan) and maintained in Dulbecco’s Modified Eagle Medium, high glucose (DMEM; Thermo Fisher Scientific, #41965-039) supplemented with 10% fetal bovine serum (FBS; Cytiva, #SV30160.03) at 37 °C in a humidified incubator with 5% CO_2_.

### NanoBiT based transducer proximity assays

Ligand-induced proximity between ORF74 and miniG_i_, Gβ1, GRK2/3/5/6 or β-arrestin1/2 was monitored using a NanoBiT complementation assay. HEK293A cells were transiently co-transfected in suspension using FuGENE® HD Transfection Reagent (Promega, #E2312) with the following biosensor pairs at a 1:1 ratio: ORF74-SmBiT with LgBiT-miniG_i_ or LgBiT-GRK2/3/5/6; and ORF74-LgBiT with SmBiT-Gβ1 or SmBiT-β-arrestin1/2. Cells were seeded in poly-D-lysine-coated white 96-well plates (Corning, #3610; 2.5 × 10^4^ cells/well; 50 ng DNA per well) and cultured for 48 h. Next, cells were washed once with assay buffer [Hanks’ Balanced Salt Solution (HBSS; Thermo Fisher Scientific), 20 mM HEPES, 0.5% FBS, pH 7.4] and incubated with 80 μL/well of a 1:200 diluted Nano-Glo® Live Cell Substrate (Promega, #N2012) working solution for 30 min at 37 °C. Luminescence was recorded using the FLIPR Penta system (Molecular Devices), with baseline recording followed by addition of serial chemokine dilutions (final concentration range, 0.06–250 nM) and monitoring for 20 min.

Real-time data were normalized to baseline and corrected by subtracting the vehicle control. Responses were quantified as area under the curve (AUC) over 0–20 min after chemokine addition, with signal changes above and below baseline defined as positive and negative responses, respectively. Data are expressed as normalized AUC (%), with the maximal CXCL1 response within each assay set to 100% for positive responses and −100% for negative responses.

### Data analysis and ligand bias assessment

Data are reported as mean ± SEM from three or five independent experiments, each performed in duplicate. NanoBiT concentration–response curves were fitted using a three-parameter logistic model in GraphPad Prism v10.4.2, from which pEC_50_ and E_max_ values were derived. For negative NanoBiT responses, E_max_ values were reported as negative values. Ligands that did not produce clear concentration-dependent responses, with absolute signal changes below 20% of the maximal CXCL1 response, were not fitted and were reported as not determined. Statistical comparisons of pEC_50_ and E_max_ values were performed using one-way ANOVA followed by Dunnett’s multiple comparisons test, with CXCL1 as the reference ligand.

Equimolar comparison plots were generated from the normalized concentration–response data by plotting responses measured at the same ligand concentrations against each other for selected pairs of ORF74-associated NanoBiT readouts. For readouts with negative NanoBiT responses, response direction was inverted for visualization and absolute E_max_ values were used for bias calculations. Ligand bias was assessed using log(E_max_/EC_50_) as a transduction coefficient, with E_max_ values expressed as fractions and EC_50_ values expressed in molar concentrations (Kolb et al., 2022).

For each readout, Δlog(E_max_/EC_50_) values were calculated by subtracting the log(E_max_/EC_50_) value of the reference ligand CXCL1 from that of each test ligand:

$$1.\;\Delta \log\left(\frac{E_{\max}}{{EC}_{50}}\right)={\log\left(\frac{E_{\max}}{{EC}_{50}}\right)}_{lig}-{\log\left(\frac{E_{\max}}{{EC}_{50}}\right)}_{ref}$$

The bias index was calculated as ΔΔlog(E_max_/EC_50_) by subtracting the Δlog(E_max_/EC_50_) value for the comparator pathway from that for the pathway of interest.

$$2.\;\Delta \Delta \log\left(\frac{E_{\max}}{{EC}_{50}}\right)={\Delta \log\left(\frac{E_{\max}}{{EC}_{50}}\right)}_{P1}-{\Delta \log\left(\frac{E_{\max}}{{EC}_{50}}\right)}_{P2}$$

Statistical significance of ligand bias was determined using multiple unpaired Welch’s t-tests with Holm–Šídák correction for multiple comparisons. Statistical significance was defined as P < 0.05.

## Results

### ELR+ CXC chemokines induce ORF74-associated transducer responses

We previously showed that ELR+ CXC chemokines interact with ORF74 with apparent potencies in the nanomolar range. CXCL1 was the most potent ligand, while CXCL2, CXCL3 and CXCL6 were only slightly less potent. CXCL5, CXCL7 and CXCL8 showed substantially lower and broadly similar potencies (Kong et al., 2025). We aimed to investigate whether these ELR+ CXC chemokines differentially engage proximal ORF74-associated transducers and thereby produce measurable ligand bias. For reference, representative raw luminescence traces for all NanoBiT biosensor pairs, recorded before and for 20 min after the addition of 250 nM CXCL1 or vehicle, are shown in Supplementary Figure S1.

Given previous evidence supporting Gi coupling by ORF74, we first monitored ligand-induced miniG_i_ recruitment as a Gi-associated activation readout. miniG_i_ is an engineered Gα_i_-derived probe that recognizes active GPCR conformations while lacking the N-terminal membrane anchors and Gβγ-binding interface of native Gα subunits (Wan et al., 2018). CXCL1–3 and CXCL6–8 induced concentration-dependent increases in ORF74–miniG_i_ luminescence, whereas CXCL5 had little effect, despite its previously observed binding to ORF74. Among the active ligands, CXCL1 and CXCL3 behaved as full agonists, whereas CXCL2 and CXCL6–8 displayed partial agonist activity. CXCL1–3 showed the highest potency, while CXCL7 was the least potent active ligand (Figure 1A; Table 1).

**FIGURE 1:**
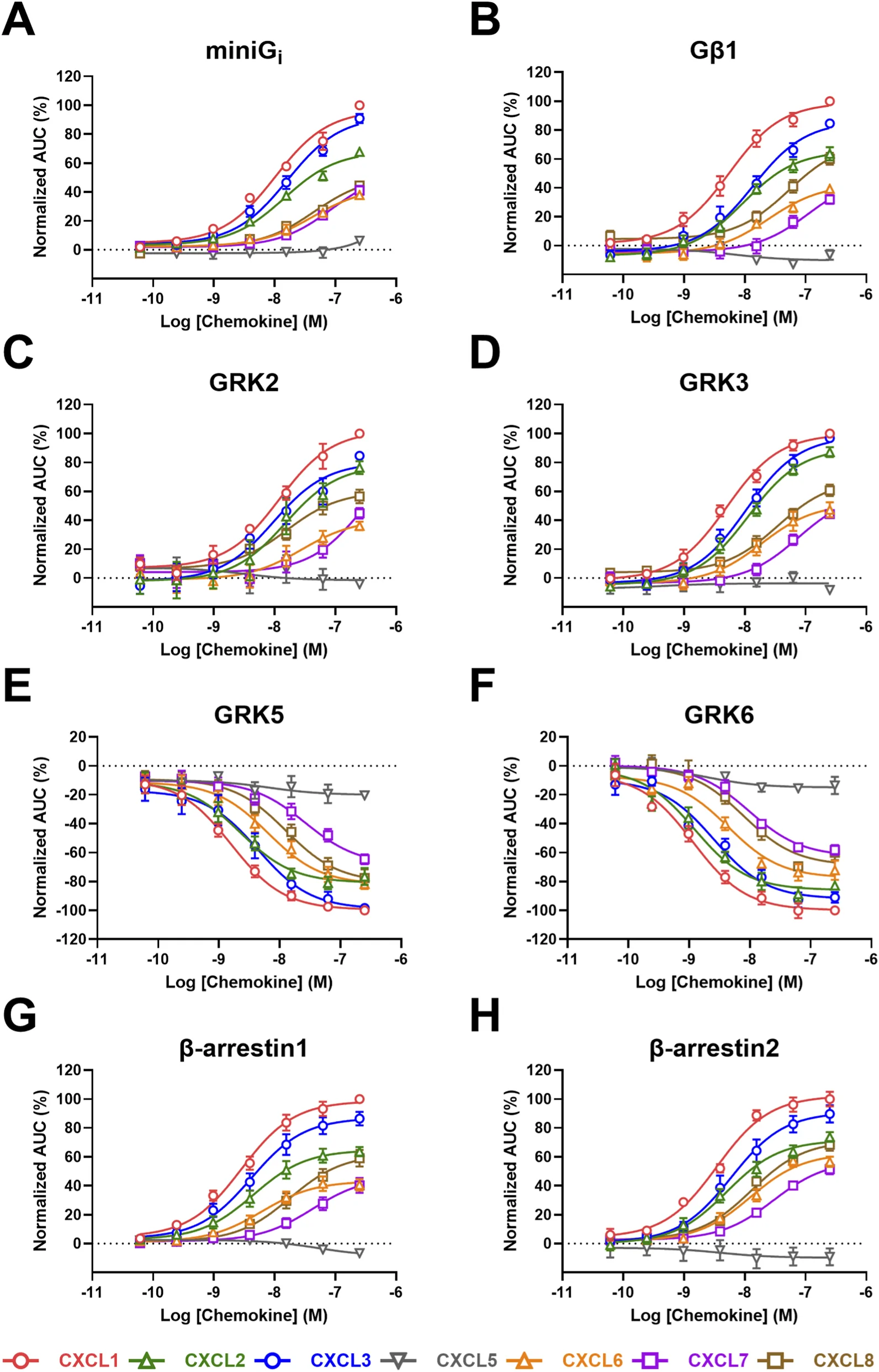
Ligand-induced ORF74-associated NanoBiT transducer responses. ELR+ CXC chemokine-induced changes in ORF74–transducer proximity were measured in HEK293A cells for **(A)** miniG_i_, **(B)** Gβ1, **(C)** GRK2, **(D)** GRK3, **(E)** GRK5, **(F)** GRK6, **(G)** β-arrestin1, and **(H)** β-arrestin2. Data are shown as normalized AUC (%), with the maximal absolute CXCL1 response in each assay set to 100%. Responses below baseline are plotted as negative values. Data are shown as mean ± SEM from five independent experiments, each performed in duplicate, except that CXCL5 was tested in three independent experiments. For β-arrestin1/2 recruitment shown in panels **(G)** and **(H)**, three independent experiments were reported previously (Kong et al., 2025), and two additional experiments were performed for CXCL1–3 and CXCL6–8 in the present study. Pharmacological parameters derived from the fitted curves are summarized in Table 1.

**TABLE 1:** Potency and efficacy of active ELR+ CXC chemokines in ORF74-associated NanoBiT readouts.

Ligand	Param.	miniG_i_	Gβ1	GRK2	GRK3	GRK5	GRK6	β-arrestin1	β-arrestin2
CXCL1	pEC_50_	7.96 ± 0.07	8.25 ± 0.15	7.91 ± 0.05	8.30 ± 0.09	8.80 ± 0.06	8.93 ± 0.07	8.53 ± 0.07	8.46 ± 0.03
	E_max_	98 ± 1	100 ± 1	103 ± 3	100 ± 2	−100 ± 2	−100 ± 3	99 ± 3	102 ± 4
CXCL2	pEC_50_	7.82 ± 0.10^ns^	8.05 ± 0.14^ns^	7.65 ± 0.07^ns^	7.96 ± 0.09^ns^	8.64 ± 0.08^ns^	8.90 ± 0.11^ns^	8.33 ± 0.10^ns^	8.29 ± 0.15^ns^
	E_max_	70 ± 3^**^	66 ± 4^****^	81 ± 5^**^	90 ± 4^ns^	−81 ± 5^*^	−86 ± 3^*^	65 ± 4^****^	72 ± 4^***^
CXCL3	pEC_50_	7.79 ± 0.13^ns^	7.98 ± 0.19^ns^	7.96 ± 0.12^ns^	7.98 ± 0.13^ns^	8.43 ± 0.17^ns^	8.60 ± 0.20^ns^	8.37 ± 0.13^ns^	8.17 ± 0.15^ns^
	E_max_	94 ± 5^ns^	86 ± 3^*^	83 ± 2^*^	100 ± 4^ns^	−100 ± 4^ns^	−93 ± 3^ns^	87 ± 5^ns^	94 ± 5^ns^
CXCL6	pEC_50_	7.46 ± 0.17^*^	7.55 ± 0.16^**^	7.62 ± 0.20^ns^	7.74 ± 0.10^**^	8.20 ± 0.17^**^	8.35 ± 0.08^**^	8.21 ± 0.17^ns^	7.91 ± 0.11^**^
	E_max_	45 ± 6^****^	46 ± 4^****^	43 ± 4^****^	52 ± 6^****^	−84 ± 5^ns^	−78 ± 6^**^	43 ± 4^****^	63 ± 3^****^
CXCL7	pEC_50_	7.06 ± 0.06^****^	6.98 ± 0.11^****^	6.81 ± 0.03^****^	7.10 ± 0.10^****^	7.58 ± 0.13^****^	8.03 ± 0.09^****^	7.35 ± 0.06^****^	7.54 ± 0.08^****^
	E_max_	56 ± 6^****^	50 ± 3^****^	70 ± 4^****^	61 ± 3^****^	−70 ± 3^***^	−63 ± 2^****^	47 ± 5^****^	58 ± 5^****^
CXCL8	pEC_50_	7.35 ± 0.11^**^	7.27 ± 0.10^***^	7.78 ± 0.20^ns^	7.45 ± 0.08^****^	7.96 ± 0.05^***^	8.12 ± 0.12^***^	7.70 ± 0.11^****^	7.86 ± 0.11^**^
	E_max_	52 ± 5^****^	74 ± 6^***^	63 ± 6^****^	69 ± 3^****^	−78 ± 6^**^	−69 ± 3^****^	63 ± 5^****^	73 ± 5^***^

Data are shown as mean ± SEM, from five independent experiments, each performed in duplicate. CXCL5 was excluded because reliable pEC_50_ and E_max_ values could not be determined. Statistical analyses were performed using one-way ANOVA, followed by Dunnett’s multiple comparisons test, with CXCL1 as the reference ligand. *P < 0.05; **P < 0.01; ***P < 0.001; ****P < 0.0001; ns, not significant.

In parallel, we monitored ligand-induced ORF74–Gβ1 proximity as a complementary readout that may reflect receptor–G protein engagement and/or conformational rearrangement (Galés et al., 2005). Similar to the miniG_i_ readout, CXCL1–3 induced robust concentration-dependent increases in ORF74–Gβ1 luminescence, whereas CXCL6–8 produced weaker responses with lower potency and/or efficacy (Figure 1B; Table 1). CXCL5 again failed to induce a clear concentration-dependent response, consistent with its minimal activity in the miniG_i_ assay (Figure 1B).

GRKs are key regulators of GPCR signaling that phosphorylate activated receptors, thereby promoting β-arrestin recruitment and subsequent receptor desensitization and trafficking. Cytosolic GRK2/3 are recruited to the plasma membrane through free Gβγ subunits released upon G protein activation, whereas the membrane-associated GRK5/6 are less dependent on Gβγ availability and can engage receptors with reduced dependence on G protein activation (Matthees et al., 2024). We next assessed how ELR+ CXC chemokines modulate ORF74–GRK2/3/5/6 NanoBiT responses.

For GRK2 and GRK3, active ELR+ CXC chemokines induced concentration-dependent increases in luminescence, with an overall pattern similar to that observed for ORF74–Gβ1. CXCL1–3 produced robust ORF74–GRK2/3 responses, whereas CXCL6–8 showed more variable and generally weaker responses (Figures 1C,D; Table 1). The response direction was reversed for GRK5 and GRK6, with chemokines producing concentration-dependent decreases in ORF74–GRK luminescence. CXCL1–3 induced substantial negative responses, whereas CXCL6–8 also produced marked decreases but with lower potency (Figures 1E,F; Table 1). Across all four GRK readouts, CXCL5 failed to induce clear dose-dependent changes in ORF74–GRK luminescence (Figures 1C–F). The negative GRK5/6 responses likely reflect ligand-induced changes in ORF74–GRK5/6 proximity and/or tag orientation rather than direct evidence of GRK5/6 dissociation from ORF74 (Palmer et al., 2022).

Finally, ligand-induced β-arrestin recruitment to ORF74 was assessed. These data include three independent experiments reported previously (Kong et al., 2025), combined with two additional independent experiments performed for CXCL1–3 and CXCL6–8 in the present analysis. CXCL1–3 and CXCL6–8 induced concentration-dependent recruitment of both β-arrestin1 and β-arrestin2, whereas CXCL5 failed to induce clear responses up to 250 nM and showed a largely neutral profile (Figures 1G,H). For β-arrestin1 recruitment, CXCL1 and CXCL3 showed the highest efficacy, whereas CXCL2 and CXCL6–8 behaved as partial agonists. A similar ligand ranking was observed for β-arrestin2 recruitment, although CXCL2 and CXCL6–8 showed higher efficacy than for β-arrestin1 recruitment (Figures 1G,H; Table 1).

### Equimolar comparison plots reveal similar ORF74-associated transducer profiles among active ELR+ CXC chemokines

To visualize how ELR+ CXC chemokines modulate ORF74-associated responses across different NanoBiT readouts, equimolar comparison plots were generated for selected pairs, with the maximal CXCL1 response set to 100% for each readout. In these plots, the dotted line indicates equal normalized responses between the two compared readouts (Rajagopal et al., 2013; Kolb et al., 2022). For CXCL1–3 and CXCL6–8, miniG_i_ and Gβ1 responses were largely aligned with the line of equal normalized response (Figure 2A). These chemokines also showed closely matched responses within the GRK2/3 and GRK5/6 subfamilies, respectively (Figures 2D,E). In contrast, the GRK3–GRK5 comparison showed a shift toward higher ORF74–GRK5 response (Figure 2F). Gβ1 responses were comparable to those of GRK3 (Figure 2G), whereas GRK5 responses were again higher in the Gβ1–GRK5 comparison (Figure 2H). Comparisons of miniG_i_ or Gβ1 with β-arrestin1/2 showed a general shift toward more pronounced β-arrestin recruitment at matched ligand concentrations (Figures 2B,C,I,J), while β-arrestin1 and β-arrestin2 recruitment was largely similar (Figure 2K). Subtle ligand-dependent differences were also observed, particularly in the miniG_i_–β-arrestin1/2 plots. In the miniG_i_–β-arrestin1 comparison, CXCL7 remained close to the line of equal normalized response, indicating similar miniGᵢ and β-arrestin1 responses, whereas CXCL1 showed a pronounced shift toward β-arrestin1 recruitment. This difference implies a relative shift of CXCL7 toward miniGᵢ compared with CXCL1 (Figure 2B). Collectively, these equimolar comparison plots indicate broadly matched responses for CXCL1–3 and CXCL6–8 in the miniG_i_–Gβ1, GRK2–GRK3, GRK5–GRK6 and β-arrestin1–β-arrestin2 comparisons, while also revealing shared readout-dependent differences in comparisons involving GRK5 or β-arrestin1/2.

**FIGURE 2:**
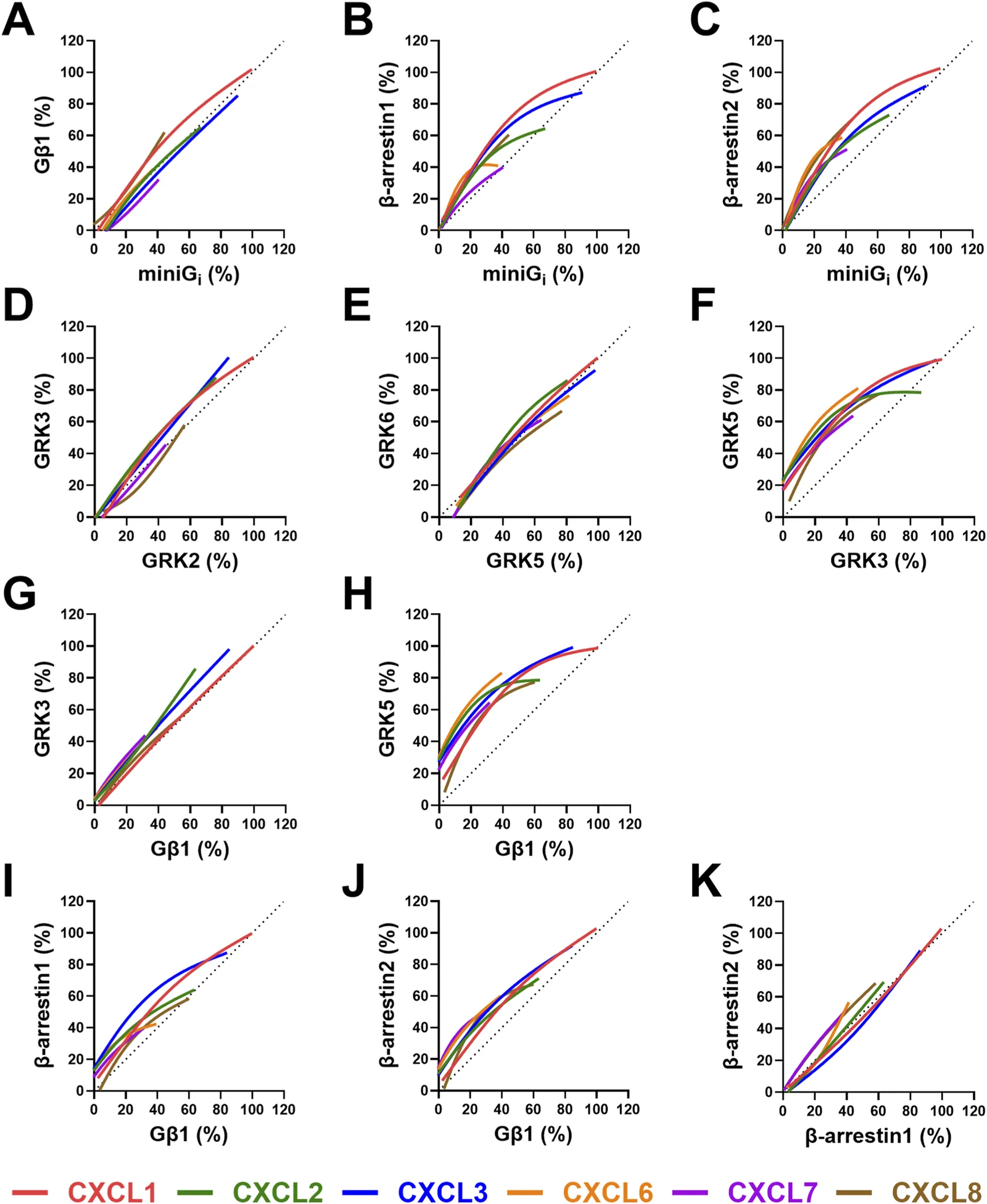
Equimolar comparison plots of ORF74-associated NanoBiT transducer readouts. **(A–K)** Normalized responses measured at the same ligand concentrations were plotted against each other for selected pairs of ORF74-associated NanoBiT readouts, with the maximal CXCL1 response set to 100% for each readout. Curves were generated from mean concentration–response values derived from five independent experiments. The dotted line indicates equal normalized responses between the two compared readouts. Only active ELR+ CXC chemokines were included. CXCL5 was excluded because reliable concentration-dependent responses could not be determined.

### Active ELR+ CXC chemokines do not display pronounced G protein-, GRK- or β-arrestin-selective bias relative to CXCL1 at ORF74

While the visual comparisons (Figures 2A–K) provided an overview of ORF74-associated transducer profiles, ligand-specific bias relative to CXCL1 was further assessed by quantitative bias index analysis. Bias index values were calculated using the ΔΔlog(E_max_/EC_50_) approach (Kolb et al., 2022), with CXCL1 as the reference ligand. For example, in the miniG_i_–Gβ1 comparison, positive values indicate preference toward miniG_i_, whereas negative values indicate preference toward Gβ1. However, no significant bias relative to CXCL1 was observed for CXCL2, CXCL3, CXCL6, CXCL7, or CXCL8 in this comparison (Figure 3A). Comparison of miniG_i_ with β-arrestin1/2 recruitment similarly revealed no major G protein-versus β-arrestin-associated bias, although CXCL7 showed a small but significant preference toward miniG_i_ over β-arrestin1 relative to CXCL1 (Figures 3B,C). We next assessed comparisons within the Gβ1–GRK–β-arrestin axis, using GRK3 and GRK5 as representative readouts for the GRK2/3 and GRK5/6 subfamilies, respectively. No significant bias was detected in comparisons of Gβ1 with GRK3/5 or β-arrestin1/2, or between GRK3 and GRK5 (Figures 3D–H). Among β-arrestin isoforms, only CXCL7 showed a small but significant preference for β-arrestin2 over β-arrestin1 (Figure 3I). Overall, in the HEK293A NanoBiT system, CXCL1–3 and CXCL6–8 displayed largely balanced engagement of ORF74 across the G protein–GRK–β-arrestin transducer axis relative to CXCL1.

**FIGURE 3:**
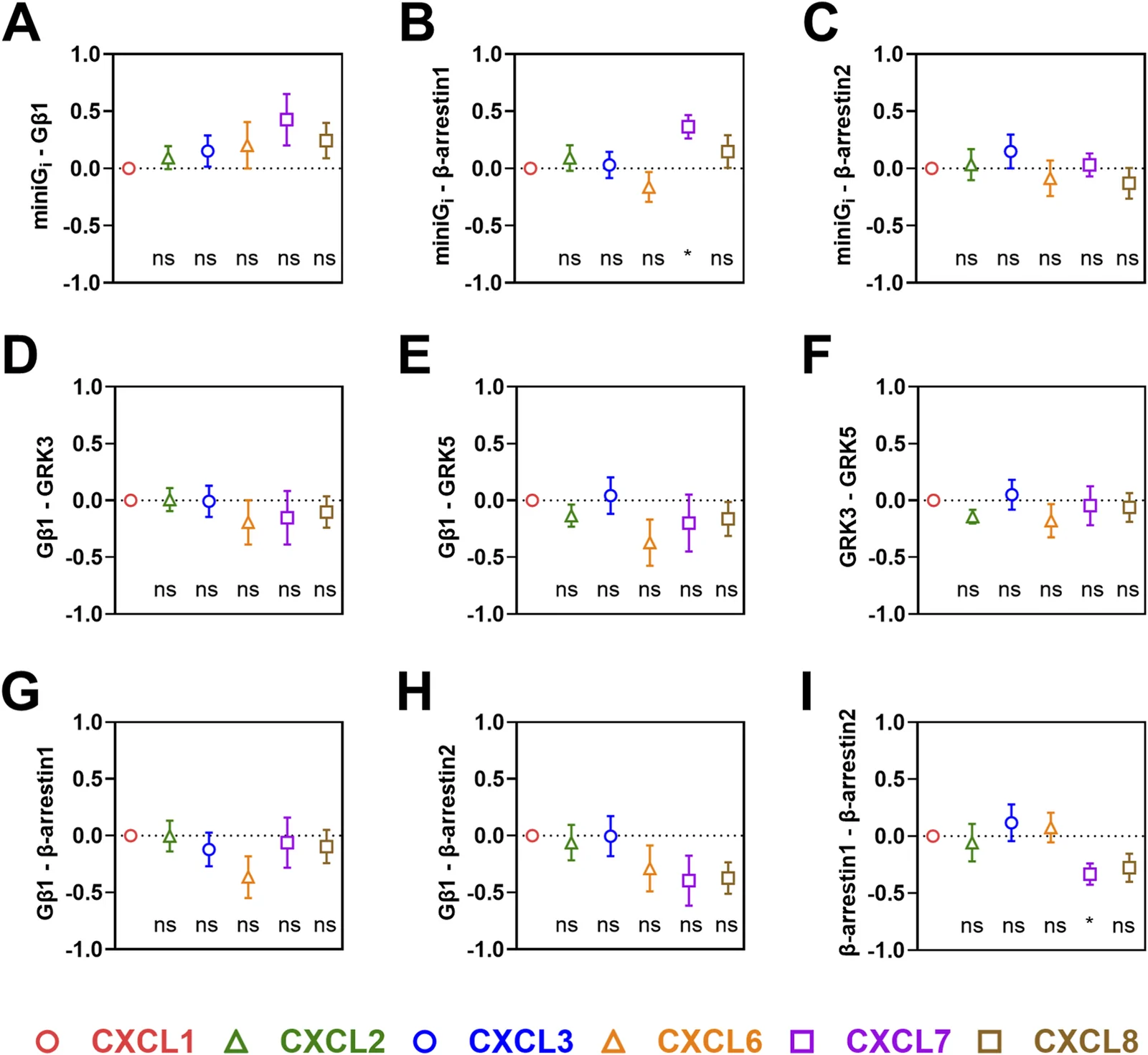
Ligand bias analysis across ORF74-associated NanoBiT transducer readouts. **(A–I)** The bias index was calculated as ΔΔlog(E_max_/EC_50_) using CXCL1 as the reference ligand, with the direction of comparison indicated by the y-axis label in each panel. CXCL5 was excluded because reliable E_max_ and EC_50_ values could not be determined by curve fitting. Data are shown as mean bias index ± SEM derived from five independent experiments. Statistical significance was assessed using multiple unpaired Welch’s t-tests with Holm–Šídák correction for multiple comparisons. *P < 0.05; ns, not significant.

## Discussion

Many human chemokine receptors interact with multiple chemokine ligands, creating a complex ligand–receptor network that regulates immune cell activation, migration, and inflammatory responses. Rather than representing simple redundancy, such promiscuous ligand recognition can generate ligand-dependent differences in receptor signaling (Eiger et al., 2021). Whether this concept can be extended to viral GPCRs remains unclear. Notably, ligand-dependent functional selectivity has been described for the human cytomegalovirus (HCMV)-encoded chemokine receptor US28, where different chemokines and engineered CX3CL1 variants differentially modulate G protein-associated calcium signaling and migration responses (Miles et al., 2018). ORF74 is another example of this complexity, since it interacts with at least fourteen chemokines, including the seven ELR+ CXC chemokines CXCL1–3 and CXCL5–8 (Rosenkilde et al., 1999; Kong et al., 2025). Previous studies have shown that these ELR+ CXC chemokines display varying degrees of ORF74 agonism, whereas other ORF74-interacting chemokines can behave as neutral ligands or inverse agonists. Moreover, pharmacological inhibitor studies showed that ORF74-mediated chemotaxis is highly Gi-dependent, whereas responses induced by individual ELR+ CXC chemokines differed in their sensitivity to Gβγ and phospholipase C (PLC) inhibition (Kong et al., 2025). These findings raised the question of whether the differences in signaling potency, efficacy, and downstream inhibitor sensitivity within this ELR+ CXC chemokine agonist series are accompanied by distinct ORF74-associated transducer interaction profiles and ligand bias.

We used NanoBiT-based proximity assays in HEK293A cells to compare how ELR+ CXC chemokines modulate ORF74 proximity to miniG_i_, Gβ1, GRK2/3/5/6 and β-arrestin1/2. Their experimental tractability and high transfection efficiency make HEK293A cells well suited for transient NanoBiT biosensor assays. By applying the same readout and cellular background across these transducer assays, this approach minimizes experimental variability and enables direct comparison of ORF74-associated transducer profiles. Overall, CXCL1–3 and CXCL6–8 induced concentration-dependent responses across multiple ORF74–transducer pairs, whereas CXCL5 failed to produce robust responses. Together with its weak agonistic activity in calcium mobilization and little to no activity in phosphatidylinositol turnover and chemotaxis (Rosenkilde et al., 1999; Kong et al., 2025), these findings suggest that CXCL5 binds to ORF74 but has only limited capacity to promote productive ORF74 signaling. Among the active ELR+ CXC chemokines, signaling potency was broadly consistent with the apparent binding potency rank order, with CXCL1–3 and CXCL6 generally showing higher potencies than CXCL7 and CXCL8. However, potency and maximal efficacy were not strictly coupled. CXCL6 produced lower maximal responses than CXCL1–3 in the NanoBiT assays, with efficacies more in line with those of CXCL7 and CXCL8.

While CXCL1-induced β-arrestin recruitment and receptor trafficking have been described for ORF74 (de Munnik et al., 2015; Kong et al., 2025), the involvement of GRKs in ORF74 regulation remains poorly understood. Previous GRK knockout studies demonstrated that GPCRs can display distinct GRK dependency for β-arrestin recruitment, with some receptors showing broad GRK2/3/5/6-mediated regulation and others preferentially depending on GRK2/3 (Drube et al., 2022). We therefore extended our NanoBiT analysis beyond miniG_i_ recruitment to the Gβγ–GRK–β-arrestin axis. To our knowledge, this is the first study to show that ELR+ CXC chemokines modulate ORF74 proximity to GRK2/3/5/6 in a ligand-dependent manner. The increased ORF74–GRK2/3 signals are consistent with ligand-induced recruitment of cytosolic GRK2/3 to activated ORF74. In contrast, the decreased ORF74–GRK5/6 signals should not be taken as direct evidence for GRK5/6 dissociation from ORF74. Additional experiments would be required to determine whether ORF74 and GRK5/6 display basal proximity before ligand stimulation. Given the distinct membrane association of GRK2/3 and GRK5/6, and the dependence of NanoBiT complementation on both protein proximity and tag orientation, reduced ORF74–GRK5/6 luminescence may instead reflect ligand-induced changes in ORF74 conformation or in the relative positioning of ORF74 and GRK5/6. This distinction is important because increasing evidence indicates that GRK subtype usage is both receptor- and ligand-dependent, influencing β-arrestin recruitment and downstream signaling (Gardner et al., 2024; Drube et al., 2022; Kawakami et al., 2022). In particular, GRK2/3-mediated β-arrestin recruitment is closely linked to G protein activation through its dependence on Gβγ, whereas GRK5/6-mediated regulation is less dependent on free Gβγ and may therefore be more compatible with β-arrestin-biased signaling (Matthees et al., 2024). In this context, recent cell-based platforms provide practical tools for profiling and screening GRK subfamily-selective inhibitor candidates (Blum et al., 2026).

The equimolar comparison plots provided an initial visual overview of ORF74-associated transducer profiles. CXCL1 showed the highest apparent binding potency and consistently robust activity across the ORF74-associated readouts examined, supporting its selection as the reference ligand for calculating ΔΔlog(E_max_/EC_50_), with concentration-response curves fitted using a fixed Hill slope of 1 (Kolb et al., 2022). However, the response profile of CXCL1 was not uniformly aligned across all readout comparisons, with visible differences observed in pairs such as miniG_i_–β-arrestin1/2, GRK3–GRK5 and Gβ1–GRK5. CXCL1 is therefore not completely unbiased across the ORF74-associated readouts, and the equimolar plots suggested that active ELR+ CXC chemokines may share qualitative pathway preferences. When quantified across selected transducer pairs, bias index values remained within ±0.5 log units, corresponding to less than ∼3-fold shifts in relative pathway preference among chemokines. Overall, although shared pathway preferences may exist, the active ELR+ CXC chemokines did not display pronounced ligand-specific bias relative to CXCL1.

Several aspects of the present study should be considered when interpreting these findings. All NanoBiT experiments were performed in HEK293A cells, whereas ORF74 signaling in KSHV-associated diseases occurs mainly in endothelial and B-cell contexts (De Groof et al., 2021; Rosenkilde et al., 2022; Tsutsumi et al., 2025). Cellular background may influence GPCR signaling readouts and the interpretation of ligand bias (Atwood et al., 2011; Vanalken et al., 2024). Similarly, altered effector expression, such as GRK expression, can influence both agonist-induced and basal receptor–β-arrestin interactions (Drube et al., 2022). In addition, receptor overexpression in engineered systems may distort apparent ligand bias (Li et al., 2023). Different LgBiT/SmBiT assignments may affect signal amplitude and complementation efficiency across biosensor pairs. However, using CXCL1 as the reference ligand within each assay for both response normalization and ligand bias calculation reduces the influence of the resulting system bias (Kolb et al., 2022). Despite these limitations, the use of a common NanoBiT platform facilitated a systematic comparison of how ELR+ CXC chemokines regulate the ORF74–Gi/Gβ–GRK–β-arrestin axis. Together, our findings indicate that the active ELR+ CXC chemokines CXCL1–3 and CXCL6–8, but not the largely neutral ligand CXCL5, engage ORF74 in a largely balanced manner across these proximal signaling readouts, thereby expanding the current understanding of ORF74 ligand pharmacology.

## Data Availability

The original contributions presented in the study are included in the article/Supplementary Material, further inquiries can be directed to the corresponding author.
