# Supplementary material for: High-affinity ELR+ CXC chemokines display balanced G protein-, GRK- and β-arrestin-associated transducer engagement at the viral G protein-coupled receptor ORF74
Source: Front Pharmacol. 2026 Sep 25;17:1953308. doi: 10.3389/fphar.2026.1953308 (PMC13649475; doi:10.3389/fphar.2026.1953308)
Supplement: Supplementary file 1 [file Image1.pdf]

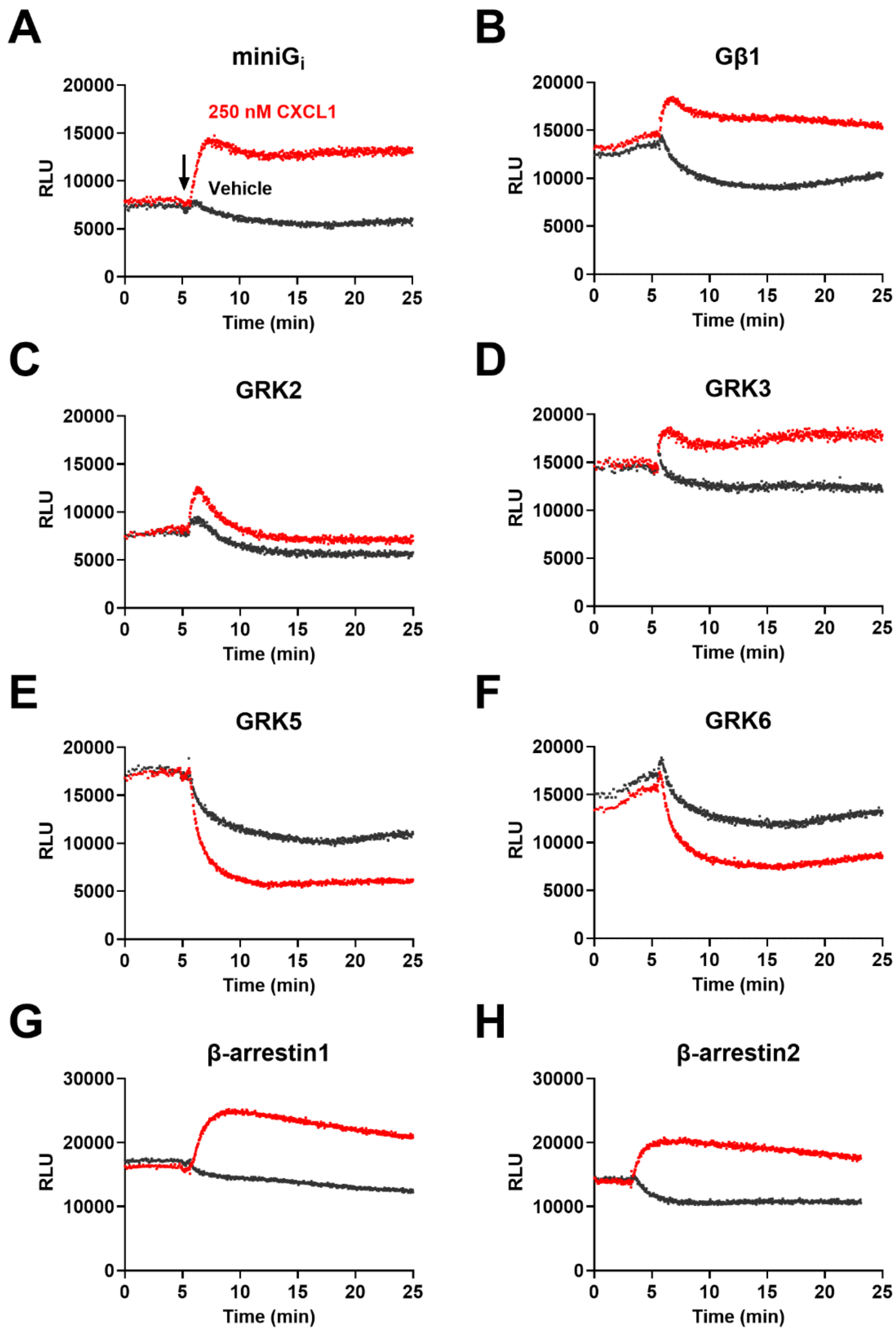

Supplementary Figure 1. Representative raw luminescence traces for ORF74-associated NanoBiT transducer responses. CXCL1-induced changes in ORF74–transducer proximity were measured in HEK293A cells for **(A)** miniG$_i$, **(B)** Gβ1, **(C)** GRK2, **(D)** GRK3, **(E)** GRK5, **(F)** GRK6, **(G)** β-arrestin1 and **(H)** β-arrestin2. Raw luminescence signals are presented in relative luminescence units (RLU) as mean values (dots) from one representative independent experiment performed in duplicate. The detector gain of the FLIPR Penta system was adjusted for each NanoBiT assay to keep the luminescence signals within an appropriate detection range. Because absolute RLU values depend on both the biosensor pair and detector gain, they should not be compared quantitatively across panels.
